# A Functional Genomic Screen Combined with Time-Lapse Microscopy Uncovers a Novel Set of Genes Involved in Dorsal Closure of *Drosophila* Embryos

**DOI:** 10.1371/journal.pone.0022229

**Published:** 2011-07-20

**Authors:** Ferenc Jankovics, László Henn, Ágnes Bujna, Péter Vilmos, Nóra Kiss, Miklós Erdélyi

**Affiliations:** Institute of Genetics, Biological Research Center of the Hungarian Academy of Sciences, Szeged, Hungary; Institut Pasteur, France

## Abstract

Morphogenesis, the establishment of the animal body, requires the coordinated rearrangement of cells and tissues regulated by a very strictly-determined genetic program. Dorsal closure of the epithelium in the *Drosophila melanogaster* embryo is one of the best models for such a complex morphogenetic event. To explore the genetic regulation of dorsal closure, we carried out a large-scale RNA interference-based screen in combination with *in vivo* time-lapse microscopy and identified several genes essential for the closure or affecting its dynamics. One of the novel dorsal closure genes, the small GTPase activator *pebble* (*pbl*), was selected for detailed analysis. We show that *pbl* regulates actin accumulation and protrusion dynamics in the leading edge of the migrating epithelial cells. In addition, *pbl* affects dorsal closure dynamics by regulating head involution, a morphogenetic process mechanically coupled with dorsal closure. Finally, we provide evidence that *pbl* is involved in closure of the adult thorax, suggesting its general requirement in epithelial closure processes.

## Introduction

Dorsal closure of the embryonic epithelium takes place during mid-embryogenesis, when two epithelial sheets migrate towards the dorsal midline where they meet and fuse [1]. The migrating epithelium is pulled by rhythmic contractions of cells in the neighboring tissue called amnioserosa. Cells of the amnioserosa progressively die by apoptosis during closure and the dorsal hole becomes sealed, generating a continuous dorsal epidermis. Other epithelial closure processes such as embryonic wound healing or closure of the adult thorax during metamorphosis, involve a coordinated series of cellular activities that are very similar to those required for dorsal closure [2]. Importantly, there is a surprisingly high degree of evolutionary conservation of mechanisms by which epithelial discontinuities are repaired, making dorsal closure of *Drosophila* an excellent model for wound healing [3].

Over the last few decades, several large-scale mutant screens have been performed to identify genes affecting embryonic morphogenesis [4]–[6]. These classical genetic screens also uncovered the roles of many genes in dorsal closure. Mutations of these genes led to the classical dorsal open phenotype: a hole in the larval cuticle. Analysis of the larval cuticle revealed that some mutants with dorsal open phenotype also exhibit defects in other morphogenetic events. Abnormalities in developmental processes such as germ band retraction or head involution, in many cases appear to be coupled with dorsal closure defects indicating close cooperation between genetic and structural elements regulating these events [7]. Genetic and cell biological characterization of the dorsal closure mutants revealed that many complex cytoskeletal rearrangements coordinated by several signaling pathways collaborate to orchestrate closure of the dorsal hole. The TGF-β/*dpp* pathway has been demonstrated to be the central element of the regulatory network of dorsal closure but JNK, *wingless, Notch* and the steroid hormone signaling pathways have also been implicated in this process [8]. In addition to the signal transduction cascades, genes encoding structural elements of the cytoskeleton and the cell adhesion complexes have been identified as being involved in dorsal closure, based on the dorsal open phenotype of their mutations [8]. Genetic and cell biological analysis revealed the involvement of several regulators of the cytoskeleton in various stages of dorsal closure. Members of the Rho, Rab and Ras GTPase families have also been implicated in the regulation of the dorsal closure [9]–[13]. In addition, three GTPase regulators, the *Rap1* activator *PDZ-GEF,* the *Rac1* activator *myoblast city* and the Rac/cdc42 repressor *rotund/racGAP84C,* were identified as participating in the complex regulation of GTPase function in the embryonic epithelium undergoing dorsal closure [14]–[16].

Although the genetics of the dorsal closure have been well explored, apparently not all components have thus far been identified**.** Despite its obvious potential as a useful model for epithelial closure processes, no systematic loss-of-function screen has been performed for genes affecting dorsal closure. RNAi has been shown to be a powerful experimental tool to efficiently silence specific genes. RNAi-based screening has been used to identify gene function systematically and rapidly in *Drosophila* and in many other organisms [17]–[21]. Therefore, we carried out a large-scale RNAi-based genetic screen to identify genes regulating embryonic dorsal closure.

It has been shown that several forces provided by various tissues contribute to dorsal closure, and loss of one of these forces can be compensated by the others [22]. In these cases the opening is closed completely, but the dynamics of the closure is abnormal. A description of these abnormalities requires a quantitative analysis of the phenotype using a mathematical model. Dynamic parameters such as length and width of the dorsal hole have to be measured and displacement velocity of the epithelium or fractional contribution of the various forces can be determined [22]. Since the previous studies used solely a dorsal opening in the larval cuticle as a phenotypic output, mutations with defects in the closure dynamics were not revealed. To overcome this limitation, we have applied a high-content screening strategy providing detailed temporal information about the dynamics of the phenotype. We combined large-scale RNAi screening with automated time-lapse video microscopy and monitored the dynamics of the closure process in living dsRNA-treated embryos.

Here we describe a genomic-scale RNAi-based loss-of-function screen for genes involved in embryonic dorsal closure. The application of automated *in vivo* video microscopy to detect phenocopies enabled us to identify genes not only essential for the closure but also genes that affect closure dynamics. We have identified novel dorsal closure genes involved in various biological processes, including small GTPase regulation, signal transduction, vesicle trafficking and embryonic patterning. Furthermore, we present a detailed cell biological analysis of the multifunctional guanine nucleotide exchange factor (GEF) *pbl*. *Pbl* affects dorsal closure dynamics both directly by regulating actin dynamics of the closing epithelium and indirectly as an essential regulator of head involution.

## Results

### RNAi screen revealed the role of six novel genes in dorsal closure

**Figure 1:**
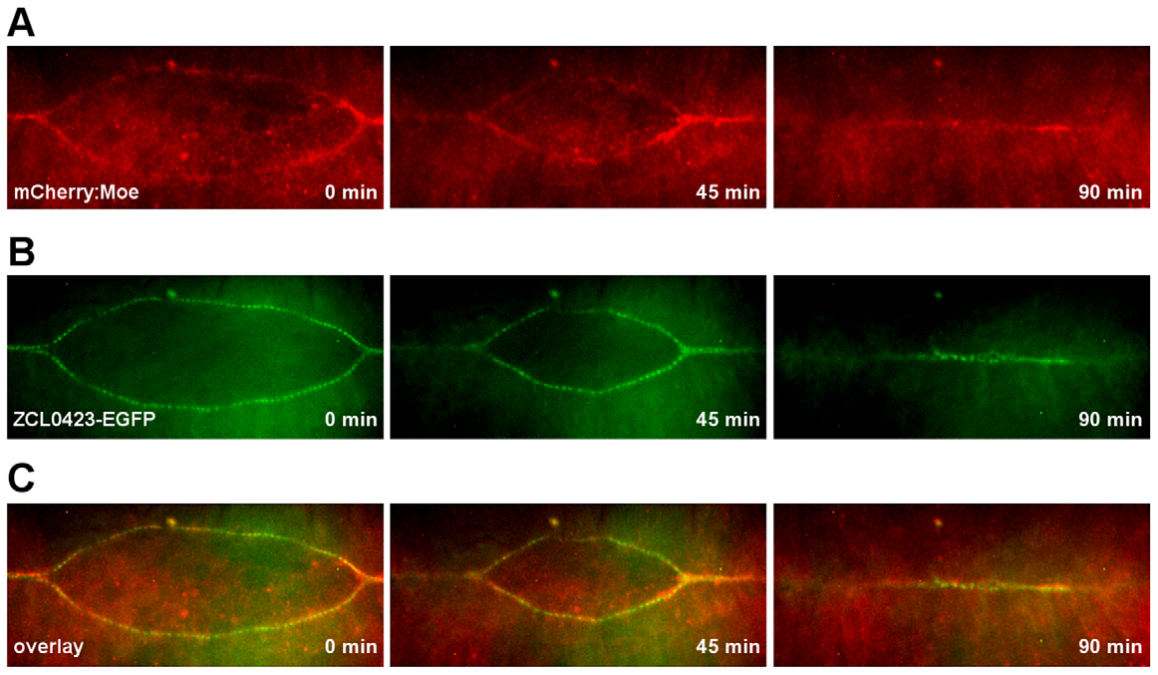
Distribution of the GFP signal in the ZCL0423 protein trap line. (A and B) Frames from movie sequences of ZCL0423/+; 69B-Gal4/UAS-mCherry:Moe embryos undergoing dorsal closure. Embryos coexpress the ZCL0423 protein trap EGFP fusion and the mCherry-tagged actin binding domain of Moesin (mCherry:Moe). (A) Expression of mCherry:Moe driven by the 69B-Gal4 driver in the epithelium highlights actin. (B) The protein trap EGFP fusion is specifically expressed in the DME cells, where it labels the leading edge. (C) Merged images, GFP in green, mCherry in red.

To identify novel genes involved in embryonic dorsal closure, we used RNAi-based genetic screening coupled with *in vivo* fluorescent video microscopy. Early *Drosophila* embryos were microinjected with dsRNAs and allowed to develop until stage 13, when germ band retraction begins. Treated embryos were then subjected to live cell imaging and the whole closure process was recorded. To visualize the leading edge, the protein trap line ZCL0423 was used which specifically labels the first row of cells in the dorsally-migrating epithelial sheets enabling easy and quick screening of the closure process [23]. In the ZCL0423 homozygous embryos, the GFP signal appeared after completion of germ band retraction in the dorsal-most epithelial (DME) cells and co-localized with the actin cables. After dorsal closure was complete, the GFP signal disappeared from the epithelial cells (Figure 1, Movie S1).

For the large-scale screening, we individually silenced a large number of genes and tested their involvement in dorsal closure by *in vivo* fluorescent confocal video microscopy. To increase the efficiency of the screening, genes were preselected which have been shown to be expressed during embryogenesis [24]. Specific dsRNAs for 2,520 genes were microinjected into embryos and time-lapse images were collected (Table S1). Initially, 32 embryos were injected with each dsRNA and on average 21 embryos were imaged *in vivo*. Image sequences were collected into movies and analyzed by visual inspection. For a more detailed analysis, in some cases the length and width of the dorsal holes were also measured. Phenotypic abnormalities were recorded into a database, the identified genes were classified by phenotypic category and the penetrance of the morphological defects was determined (Table 1).

**Table 1:** Summary of RNAi phenotypes of the identified genes.

	Gene name	Penetrance of the RNAi phenotype (%)	Biological function	Reference
Group I				
	*Bx42*	77.9±15.5	signal transduction	this study
	*CG6700*	73.6±29.6	unknown	this study
	*canoe*	85.4±10.8	cell adhesion	[28], [59]
	*Notch*	89.2±9.4	signal transduction	[27]
	*scab*	83.3±16.8	cell adhesion	[26]
	*shotgun*	92.9±8.4	cell adhesion	[29]
Group II				
	*ADP ribosylation factor 51F*	42.8±19.0	vesicle trafficking	this study
	*Krüppel*	62.4±12.9	pattern formation	this study
	*patched*	61.5±20.0	pattern formation	this study
	*pebble*	70.5±11.9	cytoskeleton regulation	this study

To increase the reliability of our screen, multiple independent tests were performed. Silencing was repeated at least two times with the dsRNAs targeting the same region of the gene product. When the penetrance of the mutant phenocopy of the injected embryos reproducibly exceeded 30% in all of the independent experiments, the gene was selected as a candidate for further analysis. By using this strategy, silencing of 12 genes resulted in abnormal dorsal closure. To avoid a potential effect of the ZCL0423 gene trap-marker in the closure process, these 12 genes were again silenced in embryos constitutively expressing the GFP-tagged actin-binding domain of Moesin (sGMCA) [25]. Live imaging of the RNAi-treated sGMCA embryos revealed that silencing of the majority of the selected genes (11/12) reproducibly caused a dorsal closure defect. To avoid false-positive results caused by off-target effects, 11 candidate genes were repeatedly silenced with dsRNAs targeting a different region of these genes. Therefore, new dsRNAs were designed, synthesized and microinjected into sGMCA embryos. Live imaging of the embryos treated with the new dsRNAs revealed that silencing of 10 out of the 11 candidate genes reproduced dorsal closure defects confirming their role in dorsal closure (Table 1). Dorsal closure defects that have not been described previously were found for six genes, whereas four genes have been previously implicated in dorsal closure.

In summary, in these series of experiments dsRNAs covering more than a third of the embryonic transcriptome were injected and a large data set of ∼60,000 time-lapse movies were produced and analyzed For the candidate genes, a large number (∼100) of embryos were injected with each dsRNA in several independent experiments and very stringent screening criteria were used. Our multiple independent RNAi-screening strategies, combined with a sensitive *in vivo* phenotyping method, uncovered a novel role for six genes in dorsal closure (Table 1).

### Group I genes are required for closure of the dorsal hole

**Figure 2:**
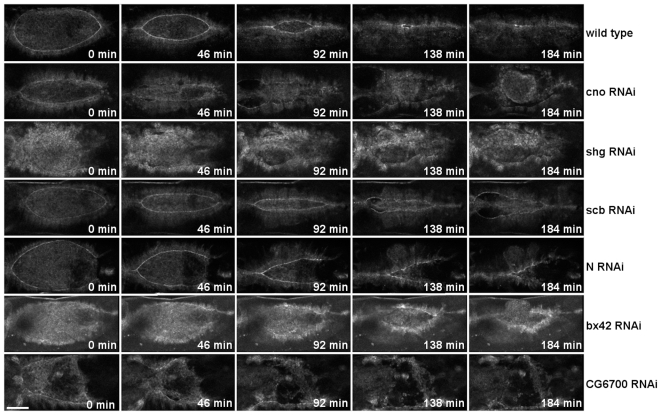
Dorsal open phenotypes generated by RNAi. RNAi phenotypes of genes in the phenotypic category I. Movie sequences show the absence of dorsal closure of dsRNA injected embryos expressing the ZCL0423 protein trap fusion protein. All embryos are shown in dorsal view with anterior to the left. Scale bar represents 50 µm.

The identified genes were grouped into two phenotypic categories. In the first phenotypic group, dorsal closure is incomplete and the dorsal hole is not closed (Group I). Silencing of *Notch (N), Bx42, shotgun (shg), scab (scb), canoe (cno)*, and *CG6700* genes resulted in this phenotype (Figure 2, Movie S2). Loss-of-function mutations in *N, shg, scb* and *cno* have previously been shown to affect dorsal closure [26]–[29]. Silencing of these genes by RNAi phenocopies the previously-described abnormalities of the loss-of-function mutants indicating the specificity of our screening approach. In this phenotypic category, in addition to the four known genes, two novel genes, *CG6700* and *Bx42,* were found to be involved in dorsal closure. Microinjection of dsRNA specific to *CG6700* resulted in a severe closure defect (Figure 2, Movie S2). Closure was initiated, the straight movement front of the epithelium was formed, the opposing sheets approached the dorsal midline but some time later closure became arrested. *CG6700* is a gene of unknown function and encodes a conserved protein containing a SAC3/GANP domain at the C-terminus. This domain has been shown to be present in proteins with diverse functions such as nuclear export factors (SAC3 of the budding yeast or mammalian GANP/MCM3-associated proteins), eukaryotic translation initiation factor 3 (eIF-3 p25), or regulators of the 26 S proteasome (Nin-1). None of these biological processes has previously been implicated in dorsal closure, thus detailed investigation of *CG6700* may reveal additional mechanisms involved in this morphogenetic event.

A similar phenotype was observed in embryos treated with *Bx42*-specific dsRNA (Figure 2, Movie S2). In these embryos, convergence of the epithelial sheets was slow and although the hole started to zipper, closure was not completed. *Bx42* encodes for a highly-conserved transcriptional regulator protein involved in various signal transduction pathways [30]. In *Drosophila*, only its involvement in Notch signaling has been demonstrated, but its vertebrate homologs interact with and modulate the activity of several other transcription factors such as Smad and steroid receptors [30]–[31]. As all of the Notch, steroid hormone and TGF-β/dpp signaling pathways are required for dorsal closure, further studies are required to determine the exact role of *Bx42* in this process [8] [32]. Since regulation of biological processes can be considered to be a combination of complex gene regulatory networks, it is tempting to speculate that *Bx42* plays a role in dorsal closure by simultaneously participating in several signaling cascades. Unfortunately, there are no loss-of-function alleles of *Bx42* available, which makes the functional analysis of this gene complicated.

### Group II genes affect dorsal closure dynamics

**Figure 3:**
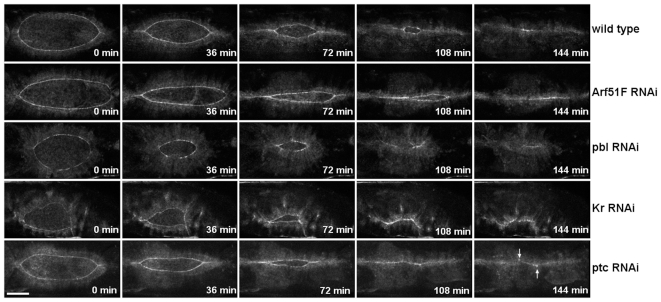
Abnormal dorsal closure dynamics generated by RNAi. RNAi phenotypes of genes in the phenotypic category II. Frames from movie sequences show abnormal dorsal closure dynamics of dsRNA-injected embryos expressing the ZCL0423 protein trap fusion protein. Arrows indicate misaligned sites. All embryos are shown in dorsal view with anterior to the left. Scale bar represents 50 µm.

Since phenotyping of the silenced embryos was performed in living embryos, we were able to identify not only genes essential for closure but also genes affecting the dynamics of closure (Group II). Accordingly, in the second phenotypic group closure took place, but with abnormal dynamics. *Krüppel (Kr), patched (ptc), ADP ribosylation factor 51F (Arf51F)* and *pbl* genes belong to this phenotypic category. Since silencing does not result in a dorsal hole in the larval cuticle, these genes have not previously been implicated in dorsal closure (Figure 3, Movie S3). However, application of our *in vivo* screening approach revealed the requirement of these novel genes in dorsal closure.

RNAi for *Kr* and *ptc* caused similar closure phenotypes. During wild-type closure the dorsal hole retains an ellipsoidal teardrop-shape throughout the entire closure process. In the embryos silenced for *Kr* and *ptc,* however, the dorsal hole is asymmetric. (Figure 3, Movie S3). In these embryos the dorsal hole is closed but a misalignment of the epithelial sheets can be detected. *Kr* is a gap gene functioning as a transcription factor, whereas *ptc* is a segment polarity gene and encodes for the Hedgehog-receptor. Both *Kr* and *ptc* are required for the patterning of the embryonic epithelium. Proper alignment of the segmented epithelium along the dorsal fusion seam requires the accurate contact of each cell with its matching cell in the opposing epithelial sheet. This remarkable accuracy of cell matching ensures the maintenance of the segmented pattern during dorsal closure [33]–[34]. Silencing of *Kr* and *ptc* disturbs segmentation which in turn, consistent with the observed phenotype, results in misalignment of the epithelial sheets.

**Figure 4:**
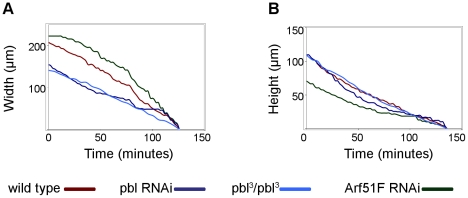
Quantification of abnormal dorsal closure dynamics. (A and B) Graphs showing closure kinetics of the dorsal hole in a buffer-injected control embryo, in a homozygous *pbl^3^* mutant embryo and embryos silenced for *pbl* and *Arf51F*. For each category, data of individual representative embryos are shown. (A) “Width” represents the maximal distance between zippering ends. (B) “Height” represents the maximal distance between the converging epithelial layers. Velocity of the epithelial sheet translocation (v), the rate constant of zippering (k_z_) and the fractional contribution of zippering (f_z_) to the velocity of the closure were calculated as described [22].

Silencing of *Arf51F* also induced abnormal closure dynamics. *Arf51F* encodes for a conserved member of the Arf family of small GTPases regulating membrane trafficking. However, the mammalian homolog of *Arf51F* (*Arf6*) has been implicated in the regulation of subcortical actin remodeling, cell adhesion dynamics and cell migration [35]–[36]. Null mutants of *Arf51F* are viable but they exhibit defects in cytokinesis in the male germ line and in the brain [37]. In addition to these phenotypes, live imaging of the embryonic morphogenesis also revealed a requirement for *Arf51F* in dorsal closure. In *Arf51F*-silenced embryos, the convergence of the lateral epithelial sheets took place normally, while zippering was inefficient in both anterior and posterior ends of the dorsal hole (Figure 3, Movie S3). As a consequence, the dorsal opening became abnormally narrow. The abnormal dynamics phenotype was characterized in a quantitative manner using a mathematical model of dorsal closure [22]. In the movies, quantitative features (height and width) of the dorsal opening were measured and the velocity of the epithelial sheet translocation (v), as well as the fractional contribution of zippering (f_z_) to the velocity of the closure were calculated. Silencing of *Arf51F* resulted in a decrease of f_z_ suggesting that *Arf51F* function is essential for efficient zippering (Figure 4, Table S2).

Suppression of *pbl* expression by RNAi also disturbed closure dynamics. The *pbl* gene encodes a guanine nucleotide exchange factor involved in the regulation of several members of the Rho GTPase family. In embryos injected with *pbl* dsRNA, the epithelial gap displayed a circular shape instead of the wild type ellipsoidal shape (Figure 3, Movie S3). The epithelial sheets converged towards the midline and the hole became sealed, however, the outline of the dorsal hole retained its roundish shape during the entire closure process. Surprisingly, by quantitative analysis of the closure dynamics in the *pbl*- silenced embryos, v and f_z_ values were found to be normal, suggesting a complex effect of *pbl* on dorsal closure (Figure 4, Table S2).

### *Pbl* and *N* are involved in both dorsal closure and the closure of the adult thorax

**Figure 5:**
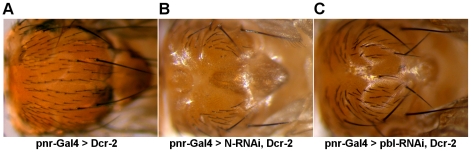
Abnormal thorax closure generated by RNAi. (A) Control thorax with pnr-Gal4/+ genotype. (B–C) Thoracic cleft phenotypes induced by pnr-Gal4-driven expression of UAS-RNAi constructs. (B) Silencing of *N*. (C) Silencing of *pbl*. The transformant ID of the UAS-RNAi constructs are KK100002 for *N* and GD35350 for *pbl*. Figures show dorsal views of adult thoraxes with anterior to the left.

Closure of the adult thorax during metamorphosis has been shown to share many signaling and structural elements with the dorsal closure of the embryonic epithelium [38]. To test the conservation of our candidate genes between these closure processes, their involvement was also tested in thorax closure. In a genome-wide screen, specific dsRNAs of all Drosophila genes has been expressed by Mummery-Widmer et al. in a tissue-specific manner at the dorsal midline during metamorphosis using the Pnr-GAL4 driver, and the loss-of-function RNAi phenotypes of the adults have been determined [39]. In this experiment – of our candidate genes – only *pbl* has been shown to be required for thorax closure. Since coexpression of *dicer2* has been demonstrated to enhance the RNAi-phenotype, we simultaneously expressed *dicer2* with dsRNAs for our candidate genes in the thorax [40]. Under these conditions, silencing of five of the ten tested genes exhibited a phenotype. Silencing of three genes (*scb, Bx42, ptc*) led to lethality, while silencing of two genes (*N* and *pbl*) caused abnormal thorax closure and resulted in the formation of a thorax cleft, suggesting the general requirement of these genes in epithelial closure processes (Figure 5).

### Pbl is involved in epithelial morphogenesis

**Figure 6:**
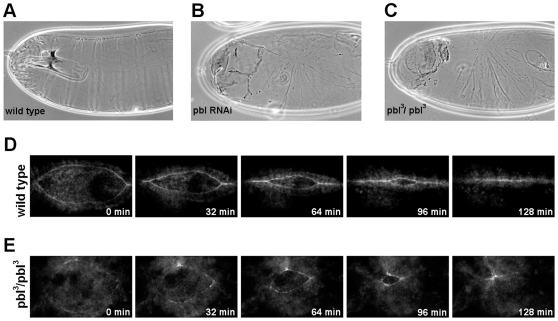
Pbl is required for the morphogenesis of the dorsal epithelium. (A–C) Cuticle preparations of embryos. (A) Wild-type cuticle. (B) Cuticle of an embryo injected with dsRNA for *pbl*. (C) Cuticle of a homozygous *pbl^3^* mutant embryo. (D and E) Frames from movies of embryos expressing ZCL0423 protein trap- EGFP fusion protein. Embryos are shown in dorsal view, scale bars represent 50 µm. (D) Wild type embryo. (E) *pbl^3^*/*pbl^3^* mutant embryo.

*Pbl*, one of the genes exhibiting thorax and dorsal closure defects, was further characterized. We investigated whether the RNAi phenotype is similar to the phenotypes of the loss-of-function *pbl* mutants by comparing the cuticles of embryos homozygous for the strong hypomorphic *pbl^3^* allele and the cuticles of *pbl* dsRNA-treated embryos. Cuticle abnormalities were detected in the *pbl* mutant embryos identical to the embryos silenced for *pbl* confirming the accuracy of our screening method. In most of the embryos (88%, n = 24) injected with *pbl* dsRNA, a serious disturbance of the epithelial matching was observed. Instead of the segmentally repeated rows of cuticle hairs present in wild-type embryos, the *pbl*-silenced embryos displayed disorganized rows of hairs meeting at one point around the dorsal midline (Figure 6A–C). We detected identical segmental misalignments in all of the homozygous *pbl^3^* mutant embryos. In addition, live imaging of *pbl* mutants expressing EGFP specifically in the DME cells revealed defects of closure dynamics identical to the *pbl*-silenced embryos (Figure 6D,E). In *pbl* mutants, quantitative parameters of the closure were similar to that of *pbl*-silenced embryos indicating further that the RNAi phenotype precisely phenocopies the loss-of-function phenotype of the *pbl* gene (Figure 4, Movie S4, Table S2).

### Pbl is involved in actin dynamics of the DME cells

*Pbl* functions as a multifunctional RhoGEF involved in the regulation of Rho and Rac GTPases [41]–[42]. *Rho1* and *Rac* genes have been shown to play a role in several aspects of dorsal closure involving amnioserosa cell contraction and dynamic cytoskeletal rearrangements in DME cells [43]–[45]. It has been shown that several forces exerted by the epithelium and the amnioserosa contribute to dorsal closure [22]. Therefore, the function of *pbl* was tested both in epithelial and in amnioserosa cells.

**Figure 7:**
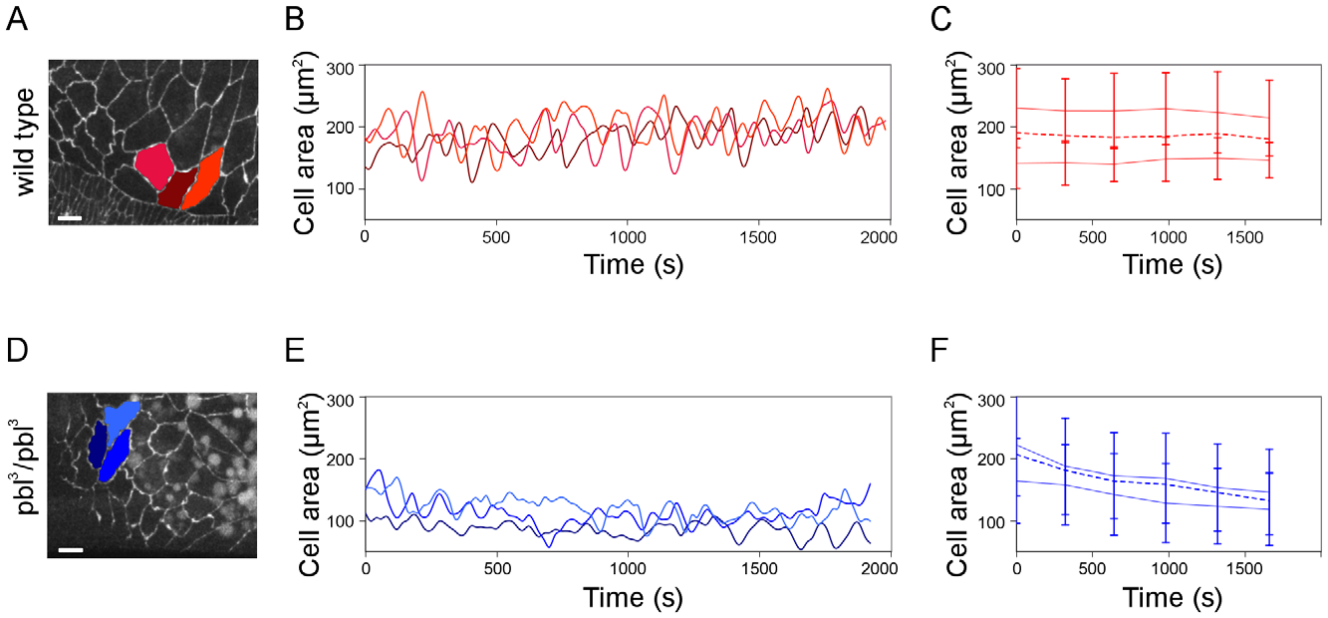
Pulsative behavior of the amnioserosa cells in *pbl* mutants. (A and D) Frames from movies of arm:GFP-expressing embryos. Scale bars are 10 µm. (B and E) Graphs showing amnioserosa cell surface fluctuations of the cells highlighted in A and D. (C and F) Mean of the apical surface maxima and minima for amnioserosa cells. Dashed lines represent average surface area of the cells (n = 18 cells in 3 embryos for wild type and n = 17 cells in 3 embryos for *pbl^3^*/*pbl^3^* mutant). (A,B,C) Wild-type control embryo. (D,E,F) *pbl^3^*/*pbl^3^* mutant embryo.

**Figure 8:**
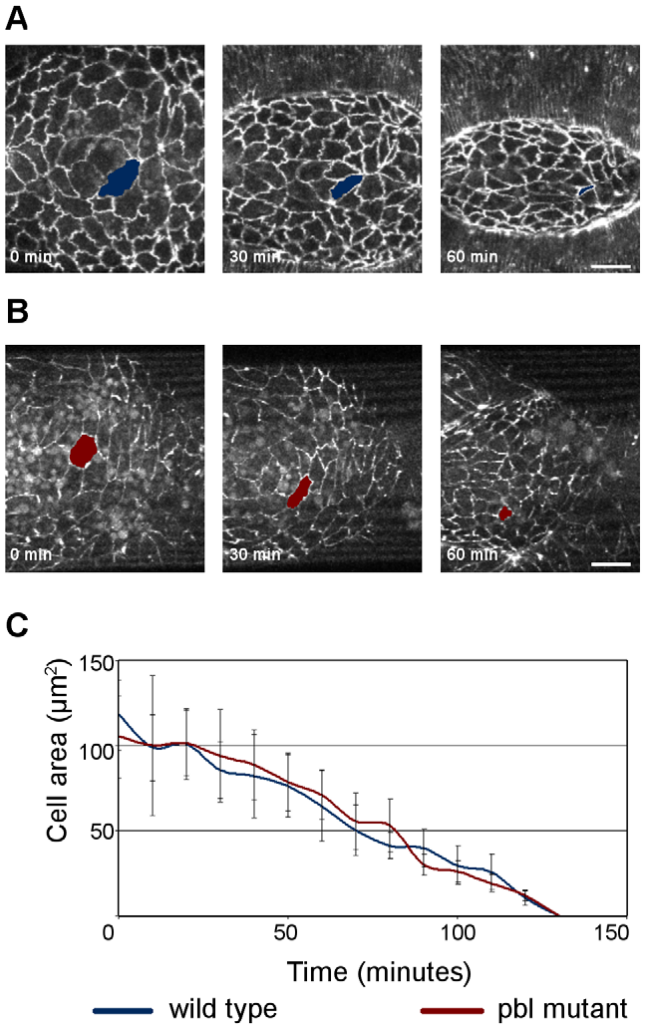
Amnioserosa dynamics in *pbl* mutants. (A and B) Frames from movies of arm:GFP-expressing embryos. Dorsal view is shown, scale bars are 20 µm. (A) Wild-type embryo. (B) *pbl^3^*/*pbl^3^* mutant embryo. (C) Quantification of amnioserosa cell contraction in a wild-type control embryo (n = 22 cells in 7 embryos) and a *pbl^3^*/*pbl^3^* mutant embryo (n = 24 cells in 6 embryos). Bars indicate standard deviation.

The closure phenotype in *pbl*-silenced and *pbl* mutant embryos is reminiscent of cases where the amnioserosa contraction is disturbed either by genetic methods or by laser ablation [22]. This similarity suggests an important role for *pbl* in the coordination of amnioserosa contraction. The apical surface of the wild type amnioserosa cells pulsate rhythmically contributing to dorsal-ward displacement of the epithelial sheets [46]. *In vivo* examination of amnioserosa cell activity of the *pbl^3^* homozygous embryos revealed that the mutant amnioserosa cells contracted and relaxed periodically but more frequently and with lower amplitude than wild type cells. In the *pbl* mutants, the periodicity of the cell surface pulsations decreased from the wild type 191±77 s value to 163±71 s (n = 85 pulsations in wild type and n = 106 pulsations in *pbl* mutants) (Figure 7, Movie S5). As closure progressed, however, the amnioserosa cells decreased their apical surface area normally (Figure 8). These results indicate that loss of *pbl* function disturbs normal pulsing of the amnioserosa cells but does not severely affects the contraction of the whole amnioserosa tissue.

**Figure 9:**
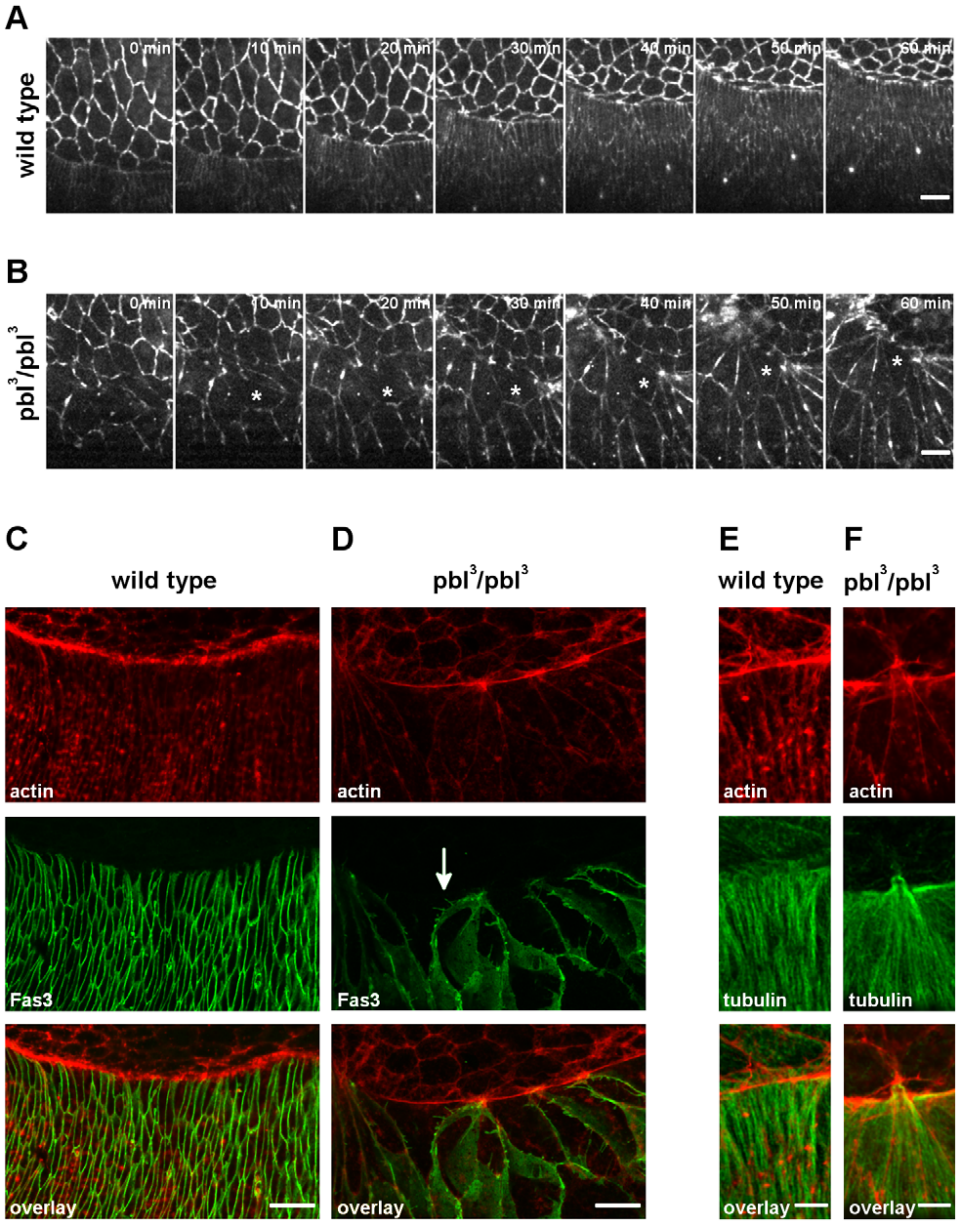
Dorsoventral polarity of the DME cells in *pbl* mutants. (A and B) Frames from movies of arm:GFP-expressing embryos. Dorsal view is shown, scale bars are 10 µm. (A) Wild-type embryo. (B) *pbl^3^/pbl^3^* mutant embryo. Asterisk labels the same cell, progressively elongating along the D/V axis. (C–F) Immunofluorescence staining of DME cells in embryos at dorsal closure stage with anti-Fas3 (green in C and D) and anti-tubulin antibody (green in E and F). Phalloidin staining of actin in DME cells (red in C–F). White arrow indicates the intrusions of the basolateral membrane. Maximum intensity projections of optical sections encompassing the whole cell volume are shown. Scale bars represent 10 µm in C and D and 5 µm in E, F. (C and E) Wild-type embryos. (D and F) *pbl^3^/pbl^3^* mutant embryos.

As other forces contributing to dorsal closure are represented by the actomyosin contraction and zippering of the DME cells, the role of *pbl* was also tested in the epithelium. *In vivo* time-lapse imaging of epithelial cells was performed in *pbl* mutants expressing arm:GFP (Figure 9, Movie S6). Consistently with previous studies, we found that epithelial cells in the *pbl* mutants were larger than in the wild type embryos because of the earlier effect of *pbl* on cell division [47]. In addition, the shape of the *pbl* mutant epithelial cells was abnormal: several DME cells were detected which were transiently elongated along the anterior-posterior body axis. However, during dorsal closure progression, these cells elongated along their dorsal-ventral (D/V) axis and adopted an approximately wild-type shape. As polarization of the DME cells along the D/V axis is an essential step in dorsal closure, we tested whether the abnormal cell size and shape was linked to abnormal D/V polarity. It has been shown that Fasciclin3 (Fas3) is excluded from the leading edge, whereas microtubules of the DME cells are arranged in parallel bundles along the D/V axis [48]–[49]. We found that in the *pbl* mutants the microtubule distribution was similar to the wild type and Fas3 was excluded from the leading edge indicating that *pbl* is not required for the D/V polarization (Figure 9). Interestingly, immunostaining of Fas3 revealed that the epithelial cells had an abnormal basolateral cell cortex. We detected long intrusions at the lateral membranes of the pbl mutant DME cells (Figure 9).

Dynamics of the actin network at the leading edge of the DME cells is critical in dorsal closure. Epithelial cells accumulate actin and extend actin-rich protrusions at their dorsal surface which have been shown to be required for normal dorsal closure. Regulation of actin accumulation and the dynamics of the extensions depend on Rho and Rac GTPases, which are targets of *pbl* in other tissues [41] [50]–[51]. To test the involvement of *pbl* in these processes, we visualized actin in fixed *pbl* mutant embryos by phalloidin staining. At the leading edge of the *pbl* mutant DME cells, a slight reduction of actin accumulation was detected (Figure 9).

**Figure 10:**
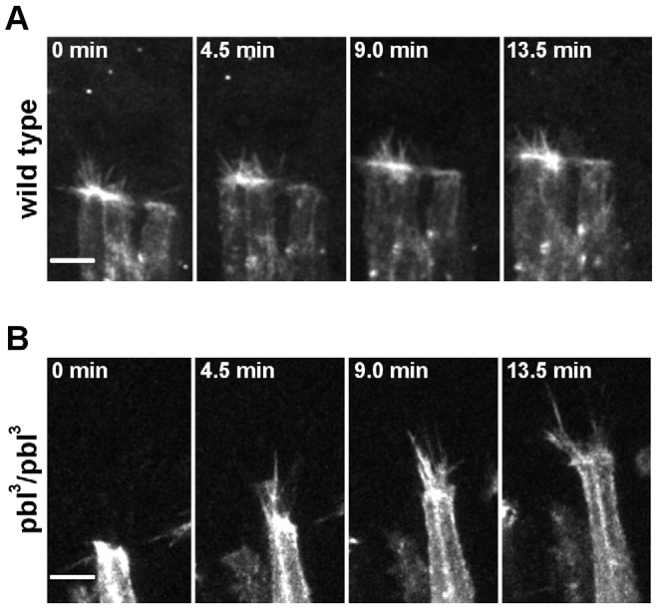
Protrusion dynamics in the DME cells of *pbl* mutants. (A and B) Frames of movie sequences showing DME cell protrusion dynamics in embryos expressing mCherry:Moe in *engrailed*-expressing cell stripes. Scale bars are 5 µm. (A) en-Gal4, UAS-mCherry:Moe control embryo. (B) Homozygous en-Gal4, UAS-mCherry:Moe; *pbl^3^* embryo.

To test whether *pbl* is involved in the regulation of protrusion dynamics at the leading edge of the DME cells, *in vivo* time-lapse imaging of the *pbl* mutant embryos was performed. The actin-rich structures were visualized by expression of Moe:mCherry in the dorsal epithelium with the en-GAL4 driver. In agreement with the hystochemical observations, a weak accumulation of actin was detected at the leading edge of *pbl* mutant DME cells (Figure 10, Movie S7). During the zippering stage, both filopodia and lamellipodia were extended but the morphology of these protrusions were abnormal. In the *pbl* mutants protrusions were more extensive, filopodia were longer (4.8±1.4 µm in wild type [n = 58] versus 7.1±1.7 µm in pbl mutant embryos [n = 52]) and lamellipodia covered a larger protrusive area, reaching up to 26.6±7.3 µm^2^ (n = 12) compared to 14.5±4.0 µm^2^ (n = 13) in wild type. Despite of the abnormal protrusions in *pbl* mutants, towards the end of the closure process, DME cells engaged with cells from the opposite side and zippered the dorsal hole. These results indicate that reduction of *pbl* function affects actin accumulation and protrusion dynamics of the DME cells.

### *Pbl* function is required for head involution

**Figure 11:**
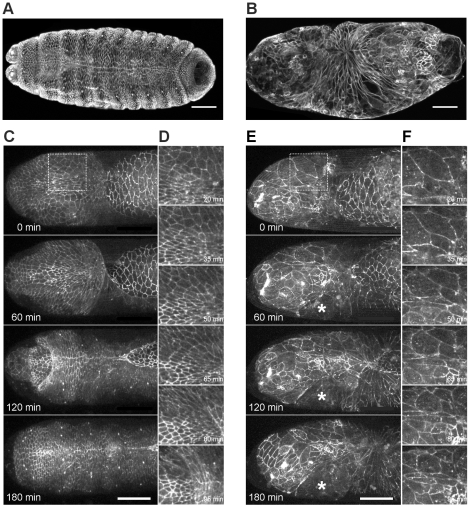
Head involution defects of *pbl* mutants. (A and B) Immunofluorescence staining of embryos after head involution stage with anti-Fas3 antibody. (A) Wild-type embryo. (B) *pbl^3^/pbl^3^* mutant embryo. (C–F) Frames from movies of arm:GFP-expressing embryos. (C) Head region of a homozygous arm:GFP embryo. (D) Enlargement of the boxed region in (C). (E) Head region of a homozygous arm:GFP; *pbl^3^* mutant embryo. Asterisk labels a rip in the head epithelium. (F) Enlargement of the boxed region in (E). (A–F) Dorsal view is shown, scale bars represent 50 µm.

It has been suggested previously that head involution, a complex morphogenetic process occurring simultaneously with dorsal closure, influences dorsal closure [7]. To analyze the correlation of these processes in *pbl* mutants, the embryonic cuticle was examined. In addition to defects in morphogenesis of the dorsal epithelium, the *pbl^3^* mutant and *pbl*-silenced embryos had abnormal head cuticles. The embryos died showing holes in the head region of the cuticle, suggesting a role of *pbl* in head involution as well (Figure 6). Immunostaining of Fas3 in the mutant embryos revealed that although the epithelial sheets met at the dorsal midline and covered the dorsal hole, the head segments of the embryo did not involute, and the head region of the embryo was not covered by epithelium (Figure 11). *In vivo* imaging of head morphogenesis in wild-type and *pbl* mutant embryos expressing arm:GFP revealed a role for *pbl* in coordinating cell shape changes in the dorsal epithelium and in the involuting tissue (Figure 11, Movie S8). In wild-type embryos, during head involution the epithelium migrates anteriorly and covers the involuting head segments. Live imaging revealed that this requires coordinated cell shape changes both in the dorsal epithelium and in the so-called acron region, the unsegmented anterodorsal part of the head. In the wild-type embryos, the epithelial cells, which became elongated along the D/V axis during dorsal closure, adopted a more cubical shape during their anterior displacement in head involution. In *pbl* mutant embryos, however, the epithelium failed to migrate anteriorly. The epithelial cells were stretched by the amnioserosa contraction pointing towards the region of the dorsal midline where closure took place, but after completion of the closure most of the epithelial cells remained elongated and did not move anteriorly (Figure 11, Movie S8).

Cells in the acron region also displayed characteristic shape changes during head involution. In the wild-type embryos, the cells at the dorsal midline became elongated along the A/P axis, whereas lateral cells elongated medio-posteriorly. As head involution proceeded, cells reduced their apical surface size and were occasionally extruded from the tissue. In *pbl* mutant embryos, a disorganized acron structure was detected. The cells were larger than in the wild type embryos and had abnormal shapes. Live imaging of the *pbl* mutant embryos revealed that, despite their morphological abnormalities, cells in the acron region were able to contract and reduce their apical surface but in an uncoordinated manner (Figure 11, Movie S8). Although some cells were stretched medio-posteriorly by the contraction of the amnioserosa, involution did not take place. Contraction and stretching of the acron cells often caused ripping of the continuous head tissue indicating defects in cell adhesion in *pbl* mutants (Figure 11, Movie S8).

In summary, our observations suggest that there are at least two causes underlying abnormal dorsal closure in *pbl* mutants. Firstly, dorsal closure defects are a direct consequence of abnormal cytoskeletal dynamics in DME cells. Secondly, dorsal closure might be indirectly affected by the abnormal head involution in *pbl* mutants suggesting a tight genetic and mechanic coupling between these two morphogenetic processes.

## Discussion

The goal of the present study was to investigate the genetic network regulating dorsal closure of the embryonic epithelium. To achieve this goal, we aimed to identify genes not previously implicated in dorsal closure. A high-throughput functional genomic screen was designed for this purpose and performed on a large scale. Our screening strategy was based on the systematic reduction of gene function by RNAi and the subsequent automated *in vivo* time-lapse imaging. This high-content assay provided both spatial and temporal information on gene activity and enabled a more comprehensive analysis of gene function. Using this approach we were able to identify not only genes essential for sealing of the epithelial sheets, but also genes which regulate the dynamics of the closure process.

In the post-genomic era, application of high-throughput RNAi has enabled the identification of gene functions at the genomic scale. Several dozen high throughput-screens have been performed on *Drosophila* and human, but these screens were typically based on cell cultures [17]. Although assay systems using cell cultures may provide valuable insights on the processes investigated, they lack the complexity of an intact developing animal and have, therefore, limited adaptability at the organism level. For example, morphogenetic movements typically require the coordinated effort of many supracellular activities, i.e., the rearrangement of the cells or the interactions of various tissues. Application of high-throughput RNAi in intact *Drosophila* embryos enabled the functional genomic analysis of such a complex developmental process as the dorsal closure of the embryonic epithelium. It has been suggested previously that the dorsal hole has to be closed in a well-defined, efficient manner [22] [48]. Defects of closure dynamics, although they do not necessarily cause morphological abnormalities, might be detrimental on an evolutionary scale. Therefore, to gain a complete overview of the genetics of dorsal closure, the presence or absence of the larval cuticle hole can not be used as the sole screening criterion. Combination of RNAi screening with time-lapse microscopy applied in this study provides temporal information on the gene function and enables the identification of genes required for the effective closure of the dorsal hole. Since the large-scale screening strategy combined with live video microscopy presented here is easily adaptable to the analysis of various embryonic developmental processes, future studies could apply it to uncover additional genes involved in morphogenesis.

Large scale automated RNAi screens tend to have low reliability. Screens using invasive dsRNA treatment methods such as microinjection of the embryo, and application of high amounts of dsRNAs run the risk of identifying many false positive hits, impeding the efficiency of further functional studies on the identified genes [20]. In order to considerably increase the specificity of our screen we applied very stringent screening criteria by performing four independent experiments and using low dsRNA concentrations. In addition, we considered only those candidates true positives that reproducibly displayed the specific phenotypes with a high penetrance in all technical repeats performed with two different gene-specific dsRNAs. As a result, ten genes have been identified as being positive hits and silencing of 26 genes known to affect dorsal closure did not result in a reproducible defect in the closure process.

In our screen, beyond the four known genes (*scb, N, shg, cno*), six novel genes were shown to influence dorsal closure. Only two of the novel genes (*CG6700* and *bx42*) have the classic dorsal-open phenotype, the complete absence of closure, while silencing of four genes (*Kr, ptc, pbl, Arf51F*) does not prevent closure but affect its dynamics. Identification of these genes demonstrates the power of the high-throughput time-lapse microscopy approach. We performed a detailed cell biological analysis of one of these genes, the multifunctional GEF, *pbl*. In this study we demonstrate its direct involvement in cytoskeletal dynamics of the dorsal epithelium and show that *pbl* indirectly affects dorsal closure dynamics by regulating head involution.

The active state of the small GTPases is controlled by guanine nucleotide exchange factors (GEFs), GTPase activator proteins (GAPs) and guanine nucleotide dissociation inhibitors (GDIs). The *pbl* gene encodes a GEF, which function as the activator of small GTPases. A remarkable feature of GEFs is that several GEFs can activate the same GTPase and several GTPases can be activated by the same GEF [52]. In addition to this obvious redundancy, most of the GEFs and target GTPases are broadly expressed and their expression patterns widely overlap. This complexity of GTPase regulation by GEFs enables a plethora of possible interactions which makes the functional analysis of the individual GEF at the organism level very complicated. Consistently, *pbl* is a pleiotropic gene required in a wide range of developmental processes [41]–[42]. *Pbl* protein has been shown to be able to activate several GTPases in *Drosophila* in a tissue and developmental stage-specific manner [41]. The essential role of *pbl* in cytokinesis and mesoderm development has been studied extensively in *Drosophila*. Since both processes require cell shape changes, *pbl* has been suggested as a component of the intracellular signaling pathway mediating cytoskeletal dynamics. *Pbl* activates *Rho1* in the contractile ring during cytokinesis in blastodermal embryos and the Rac GTPase pathway during mesoderm migration, suggesting two separate functions for *pbl* in these processes.

The mesodermal target of *pbl*, Rac, has been shown to be essential in the DME cells for normal cytoskeletal dynamics [45]. Unlike *pbl*, however, loss of Rac activity results in the absence of protrusions of the DME cells. Since the *pbl* mutant phenotype presented here is different from the Rac mutant phenotype, it is very unlikely that *pbl* activates Rac in the epithelial cells during dorsal closure. We suggest that during dorsal closure, *pbl* might activate the Rho GTPase pathway. Two lines of evidence support this hypothesis. The loss-of-function *pbl* phenotype of the DME cells very closely resembles to that of the *Rho1* mutants, both at the cellular and cuticle levels. Reduced *Rho1* or *pbl* function in the DME cells results in weak actin accumulation and excessive filopodia activity at the dorsal surface. In addition, similar to *pbl* mutants, zygotic loss of *Rho1* activity results in abnormal dorsal cuticle morphology [44] [53]. The similarity of the loss of function *Rho1* and *pbl* phenotypes in the dorsal ectoderm suggests that these genes act in the same pathway. According to our model, *pbl* activates *Rho1* in the DME cells, which in turn regulates actin accumulation and protrusion dynamics at the dorsal surface. However, the *pbl* and the *Rho1* mutant phenotypes are not completely identical [54]. This discrepancy could be explained by the presence of maternally-provided proteins or the hypomorphic nature of the mutant alleles used. A further explanation could be that *Rho1* is activated by additional GEFs or *pbl* activates additional GTPases in the DME cells.

Mutations in many genes involved in dorsal closure also result in head involution defects [7]. How loss of *pbl* activity leads to head involution defects is not completely clear. One possible function for *pbl* during head involution could be the regulation of actin dynamics in the translocating tissues through the activation of one or more GTPase pathways. This hypothesis is less attractive, since no specific actin accumulation or protrusion formation in cells of the head region has been reported so far. However, as our knowledge of the details of head involution is very poor, we can not exclude this possibility. An alternative scenario for the role of *pbl* in head involution could be that it regulates cell adhesion dynamics in the head region. Our results demonstrate that cell-cell contacts are weak in *pbl* mutants which eventually leads to ripping of the head epithelium. Activity of several GTPases of the Rho family has consistently been shown to be required for cell adhesion in a wide range of organisms and cell lines [54]–[55]. A further support for this hypothesis is that mutations of *Rho1* and RacGTPases abolish head involution [45] [53]. Further experiments are required to precisely determine the *pbl* targets in this developmental process.

Analysis of the *pbl* mutant phenotype suggests a tight mechanical connection between head involution and dorsal closure. Biophysical studies revealed the presence of a force acting on the dorsally-migrating epithelial sheets and exerted by tissues undergoing head involution [56]. This force pulls the dorsal epithelial sheets towards the anterior and provides a mechanical factor which forces the two opposing dorsal epithelial edges towards the dorsal midline thereby tightening the dorsal hole. Since *pbl* mutations abolish head involution, this force might be lost in these mutant embryos which, as a consequence, would affect closure dynamics indirectly. Moreover, additional forces generated by zippering and actomyosin contraction at the leading edge are also perturbed in *pbl* mutant DME cells. Efficient zippering requires the coordinated activity of cell extensions whereas actomyosin contraction depends on actin accumulation at the leading edge of the DME cells. Both processes are perturbed in *pbl* mutants, directly affecting closure dynamics. Thus, the only force serving dorsal closure in the *pbl* mutants is the force generated by amnioserosa contraction. We consistently detected a normal reduction of apical surface area of amnioserosa cells in *pbl* mutants as compared to wild type. Since dorsal closure is a robust process, loss of the various forces can be compensated by other tissues: the pulling force provided by the amnioserosa is sufficient to close the hole, but the dynamics of closure is abnormal.

We provide evidence that *pbl* is also required for thorax closure during metamorphosis, indicating its general role in epithelial closure processes. The requirement of several GTPases (*Rac1, cdc42, Rab11, Rab5, Rab30*) has been demonstrated in thorax closure but no function for *Rho1* has been reported so far [10] [57]. Activation of Rac occurs through the Crk–Mbc–ELMO GEF-complex, but cdc42 or Rab activation during thorax closure is still obscure. Further studies are required to determine whether additional GTPases function during thorax closure and which of these GTPases are activated by *pbl*.

## Materials and Methods

### Drosophila stocks

We used the ZCL0423 protein trap line and sGMCA:GFP to visualize the DME cells. The fly stocks en-Gal4, 69B-Gal4, pnr-GAL4, UAS-dicer2, pbl^3^ and arm:GFP were obtained from the Bloomington Stock Center. For inducible silencing of the selected genes, UAS-RNAi lines were obtained from the Vienna Stock Center. The UAS-Moe:mCherry fly stock was provided by T. Millard [33]. To analyze the actin dynamics of the epithelial cells, homozygous en-Gal4, UAS-Moe:mCherry flies were used.

### Embryo injection and RNAi screening

A commercially available dsRNA library was used for the large scale screen (Open Biosystems, [58]). To select genes expressed in the embryo, microarray data were used [24], (GEO accession number: GSE3955). For the microinjections, freshly laid homozygous ZCL0423 or sGMCA:GFP embryos were collected for 30 minutes at 25°C on juice-agar plates, washed with water and dechorionated in 50% Chlorox bleach for 2 minutes. Embryos were oriented on a juice-agar plate and transferred to a coverslip covered with glue. Embryos were desiccated and covered with Voltalef H10S halocarbon oil (VWR). Syncytial blastoderm embryos were injected laterally with dsRNAs at ca. 50% egg length. The concentration of injected dsRNA solution was ≈0.5 µg/µl in TE buffer. Microinjections were performed with glass capillaries using Transjector 5246 (Eppendorf). Capillaries were prepared with a Flaming/Brown micropipette puller P-97 (Sutter Instrument Co.). After injection, coverslips were transferred onto a home-made coverslip holder suitable for simultaneously carrying 14 coverslips.

### Time-lapse analysis

For the large-scale screen, after injection, embryos were allowed to develop to stage 13 under oil and were subsequently imaged at 25°C on an Olympus CellR fluorescent microscope equipped with a disc-scanning unit. A 10X objective and an F-View II camera (Soft Imaging System, Münster) were used for time-lapse imaging. Stage positions for each embryo were adjusted manually. Unfertilized eggs or embryos leaking cytoplasm were not imaged. Each embryo was imaged for 13 hours, and images were acquired every 12–15 minutes. Time-lapse movies for each injected embryo were stored as multi-dimensional tiff files and analyzed using ImageJ software. Publication quality images of dsRNA treated embryos were made with Leica TCS SP5 confocal microscope. DsRNA samples were coded, injections and analysis of the movies were performed blind. For the time-lapse movies of *pbl* mutants, embryos expressing arm:GFP or Moe:mCherry were imaged with an Olympus FW1000 confocal microscope. Geometric parameters of the closure were measured with ImageJ and analyzed with Microsoft Excel and DataFit. Velocity of the epithelial sheet translocation (v), the rate constant of zippering (k_z_) and the fractional contribution of zippering (f_z_) to the velocity of the closure were calculated as described previously [22].

### Immunohistochemistry

Immonostainings were performed as described earlier [49]. Primary antibodies used were anti-Tubulin (1∶100, Sigma) and anti Fas3 (1∶50, DSHB). To stain actin, embryos were incubated for 2 hrs in rhodamin-phalloidin (2 unit/ml in PBT, Molecular Probes). Specimens were mounted in 50% glycerol/PBS and examined with an Olympus FW1000 confocal microscope. Z-stacks of optical sections were recorded, maximum intensity projections of the optical sections were made with ImageJ, intensity values and color balance were adjusted with GIMP software.

## Supporting Information

Movie S1 **Distribution of the EGFP signal in the ZCL0423 protein trap line.** Movie shows dorsal closure of an embryo simultaneously expressing ZCL0423-EGFP and mCherry-tagged actin binding domain of Moesin (mCherry:Moe). Moe is shown at the top, ZCL0423 in the middle and the overlay at the bottom with mCherry:Moe in red and ZCL0423-EGFP in green. The mCherry:Moe highlights actin and the protein trap EGFP fusion labels the leading edge of the DME cells.
(MOV)

Movie S2 **Dorsal open phenotypes generated by RNAi.** Movies show the absence of dorsal closure of dsRNA injected embryos expressing the ZCL0423-EGFP protein trap fusion protein. Scale bar represents 50 µm.
(MOV)

Movie S3 **Abnormal dorsal closure dynamics generated by RNAi.** Movies show abnormal dorsal closure dynamics of dsRNA-injected embryos expressing the ZCL0423-EGFP protein trap fusion protein. Scale bar represents 50 µm.
(MOV)

Movie S4 **Dorsal closure in** ***pbl*** **mutant embryos**. Movies show a dorsal view of convergence and zippering of the two opposite epithelial cell sheets. The leading edge of the DME cells is highlighted by the ZCL0423 protein trap. Scale bar is 50 µm. The movie on the left shows normal closure in a wild type control embryo and the movie on the right shows dorsal closure of a *pbl^3^/pbl^3^* mutant embryo.
(MOV)

Movie S5 **Pulsative behavior of the amnioserosa cells in** ***pbl*** **mutants.** Pulsation of the amnioserosa cells in wild-type control and in *pbl* mutant embryos is shown. The cells are outlined by arm:GFP. Scale bars are 10 µm. The movie on the left shows amnioserosa cells in a wild type control embryo and the movie on the right shows amnioserosa cells of a *pbl^3^/pbl^3^* mutant embryo.
(MOV)

Movie S6 **Cell shape changes in** ***pbl*** **mutant embryos.** Epithelial cells expressing arm:GFP are shown in embryos undergoing dorsal closure. Scale bars are 10 µm. The movie on the left shows elongation of epithelial cells in a wild type control embryo and the movie on the right shows elongation of epithelial cells in a *pbl^3^/pbl^3^* mutant embryo.
(MOV)

Movie S7 **Protrusions of DME cells in** ***pbl*** **mutant embryos.** Movie sequences of protrusions forming at the leading edge of the DME cells are shown. Only engrailed expressing epithelial cells are visible due to en-Gal4 driven mCherry:Moe expression. Scale bars are 5 µm. The movie on the left shows cell protrusions in a wild type control embryo and the movie on the right shows protrusions in a *pbl^3^/pbl^3^* mutant embryo.
(MOV)

Movie S8 **Head involution defects of** ***pbl*** **mutants.** Movies show a dorsal view of head involution of embryos expressing arm:GFP. The movie on the left shows head involution of a wild type control embryo and the movie on the right shows a *pbl^3^/pbl^3^* mutant embryo unable to undergo head involution. Enlargements of the boxed regions show cells shape changes of the wild type and the *pbl* mutant embryo.
(MOV)

Table S1 **List of genes tested by dsRNA microinjection.** Table shows the genes targeted by RNAi, position in the dsRNA library and dsRNA concentration.
(XLS)

Table S2 **Quantitative parameters of dorsal closure of individual embryos.** Table shows quantitative parameters of closure dynamics in buffer-injected control embryos, in homozygous *pbl^3^* mutant embryos and embryos silenced for *pbl* and *Arf51F*. Velocity of the epithelial sheet translocation (v), the rate constant of zippering (k_z_) and the fractional contribution of zippering (f_z_) to the velocity of the closure are shown.
(XLS)
